# Screening of Indanoyl-Type Compounds as Elicitors of Isoflavonoid Phytoalexins in Colombian Common Bean Cultivars

**DOI:** 10.3390/molecules27113500

**Published:** 2022-05-30

**Authors:** Diego Aristizábal, Jesús Gil, Winston Quiñones, Diego Durango

**Affiliations:** 1 Escuela de Química, Facultad de Ciencias, Universidad Nacional de Colombia-Sede Medellín, Carrera 65, Medellín P.O. Box 3840, Colombia; daarist1@unal.edu.co; 2 Departamento de Ingeniería Agrícola y Alimentos, Facultad de Ciencias Agrarias, Universidad Nacional de Colombia-Sede Medellín, Carrera 65, Medellín P.O. Box 3840, Colombia; jhgilg@unal.edu.co; 3 Grupo de Química Orgánica de Productos Naturales, Instituto de Química, Universidad de Antioquia, Calle 70, Medellín P.O. Box 1226, Colombia; wiston.quinones@udea.edu.co

**Keywords:** *Phaseolus vulgaris* L., elicitor, phytoalexin, phaseollin, phytotoxicity, anthracnose

## Abstract

Eleven indanoyl derivatives were synthesized and, along with methyl jasmonate, evaluated as isoflavonoid-phytoalexin elicitors in two cultivars of common bean (*Phaseolus vulgaris* L. cvs. ICA-Cerinza and Uribe Rosado, tolerant and susceptible to anthracnose, respectively). Indanoyl derivatives (an ester, two amides, and eight indanoyl-amino acid conjugates) were obtained from 1-oxo-indane-4-carboxylic acid. In general, the accumulation of isoflavonoid-type phytoalexins, such as isoflavones (genistein, daidzein, and 2′-hydroxygenistein), isoflavanones (dalbergioidin and kievitone), isoflavan (phaseollinisoflavan), coumestrol, and pterocarpans (phaseollidin and phaseollin), was dependent on the common bean cultivar, the post-induction time, and the elicitor structure. Isoflavones, dalbergioidin, and coumestrol reached their highest amounts during the first 48 to 72 h, whereas kievitone, phaseollinisoflavano, and the pterocarpans reached maximum levels between 72 and 96 h. The 1-oxo-indanoyl-L-isoleucine methyl ester elicited the highest levels of phytoalexins (similar to those elicited by the methyl jasmonate) and showed no significant phytotoxic effects on common bean seedlings. The indanoyl-type synthetic elicitor, 1-oxo-indanoyl-L-isoleucine methyl ester, may represent a promising agronomic alternative for disease control in common bean by enhancing the accumulation of antimicrobial isoflavonoid phytoalexins.

## 1. Introduction

Plants often respond to microbial infection by producing antimicrobial, low-molecular-weight secondary metabolites known as phytoalexins. These compounds also accumulate in plants in response to various agents called elicitors, including substances of biotic (from pathogens or the same plant) and abiotic origin, such as synthetic compounds. The exogenous application of elicitors allows for an increase the phytoalexin concentration and, consequently, enhances the resistance of plants to fungal infections [1]. Therefore, elicitors have been postulated as a friendly alternative to fungicides due to their non-biocidal character. Unfortunately, very frequent applications or in applications in high concentrations of some synthetic elicitors may result in phytotoxicity symptoms [2,3].

**Figure 1:**
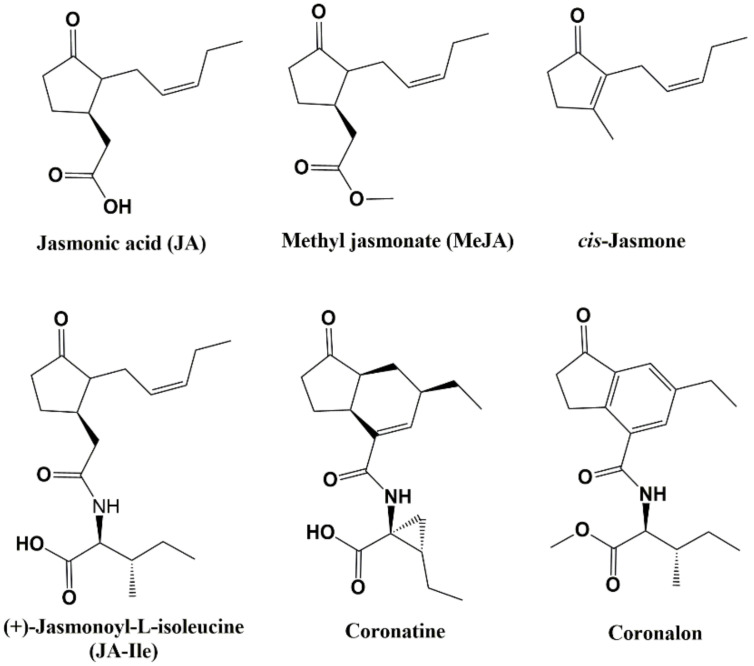
Structure of jasmonate-type elicitors and structurally-related compounds.

Exogenous application of jasmonate-type elicitors, which are biologically active fat-derived cyclopentanones present in the plant kingdom, such as jasmonic acid (JA) (Figure 1) and methyl jasmonate (MeJA), have been reported to activate the hyperproduction of various secondary metabolites [4,5,6]. In addition, coronatine, a bacterial toxin produced by *Pseudomonas syringae* that mimics the plant hormone jasmonoyl-L-isoleucine, together with some structurally-related compounds (such as indanoyl-amino acid conjugates) elicits secondary metabolite accumulation in both plant tissues and cell cultures [7,8]. Thus, indanoyl-type compounds, including coronalon (a synthetic 6-ethyl indanoyl isoleucine conjugate) have demonstrated elicit volatile compounds in Lima bean (*Phaseolus lunatus* L.) [9] and tobacco (*Nicotiana tabacum* L.) [10], anthocyanins in grapevine (*Vitis vinifera* L.) [11], and phytoalexins in rice leaves (*Oryza sativa* L.) [12] and common bean (*Phaseolus vulgaris* L.) [13], among others. The induction of plant stress responses and simple and efficient synthesis suggest that indanoyl-type compounds might be employed in agriculture to elicit plant resistance against pathogenic micro-organisms [8].

On the other hand, the common bean (*Phaseolus vulgaris* L.) is an important legume crop around the world, with an estimated annual production of around 12 million tons [14]. It represents the main source of protein for nearly five hundred million people in Africa, Latin America, and the Caribbean [15]. Additionally, common bean has a high content of dietary fiber, complex carbohydrates, vitamins, minerals, and phytochemicals [16]. Unfortunately, the high incidence of diseases and the use of susceptible cultivars limit the production of common beans. In South and Central America, the most limiting disease of the common bean is anthracnose caused by the fungus *Colletotrichum lindemuthianum* Sacc. and Magnus. Traditionally, the disease is controlled using fungicides, some of which have negative effects on the environment and humans due to their low selectivity and biocidal character [17]. In this sense, the elicitation of defensive responses in common bean emerges as a secure and ecofriendly alternative for replacing the current fungicides.

**Figure 2:**
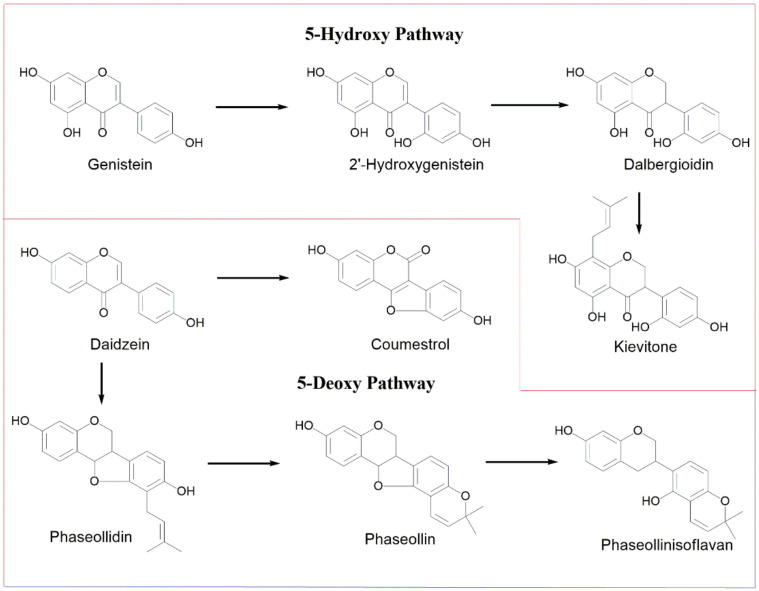
Biosynthetic pathways of isoflavonoid phytoalexins in common bean (*P. vulgaris* L.).

Elicitation of common bean increases the levels of isoflavonoid-type phytoalexins; two separate biosynthetic pathways have been proposed: 5-deoxy- and 5-hydroxyisoflavonoid (Figure 2). The first pathway involves the conversion from daidzein to coumestrol or to pterocarpan-type phytoalexins (phaseollidin and phaseollin) and phaseollinisoflavan [13]. The 5-hydroxyisoflavonoid route passes from genistein to 2′-hydroxygenistein, then dalbergioidin and, finally, kievitone. The aim of the present work was to analyze the isoflavonoid-type phytoalexin elicitor effect of a series of 1-oxo-indane-4-carboxylic acid derivatives (indanoyl derivatives) in two Colombian common bean varieties (ICA-Cerinza and Uribe Rosado). Then, the effect of post-induction time and phytotoxicity for the most active derivative as elicitor was evaluated.

## 2. Results

### 2.1. Influence of Indanoyl-Derivative Structure on Phytoalexin Accumulation

**Figure 3:**
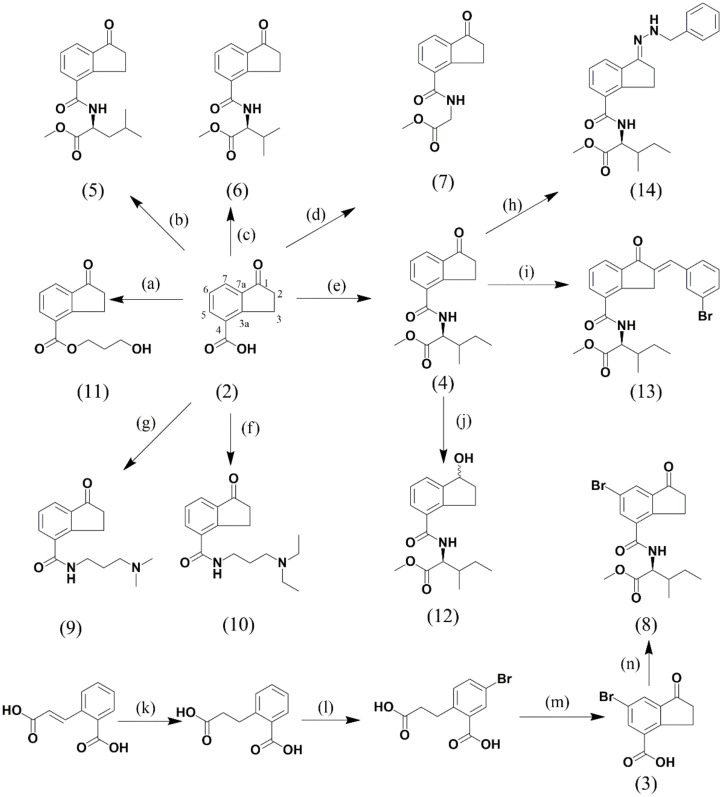
Synthesis and structures of the synthesized indanoyl derivatives. (**a**) Compound (**2**)/acetone, K_2_CO_3_, 2 h, then 3-bromo-1-propanol, 60 °C. (**b**) Compound (**2**)/DCM, HOBt, DMF, L-leucine methyl ester. (**c**) Compound (**2**)/DCM, HOBt, DMF, L-valine methyl ester. (**d**) Compound (**2**)/DCM, HOBt, DMF, glycine methyl ester. (**e**) Compound (**2**)/DCM, HOBt, DMF, L-isoleucine methyl ester. (**f**) Compound (**2**)/DCM, HOBt, DMF, 3-(diethylamino)-1-propylamine. (**g**) Compound (**2**)/DCM, HOBt, DMF, 3-(dimethylamino)-1-propylamine. (**h**) Compound (**4**)/ethanol, benzylhydrazine, reflux, 24 h. (**i**) Compound (**4**)/piperidine, 3-bromobenzaldehyde, r.t, 12 h. (**j**) Compound (**4**)/MeOH/DCM, NaBH_4_, r.t, 2 h. (**k**) 2-Carboxycinamic acid/AcOH, Pd/C, H_2_, 24 h rt. (**l**) AcOH (65%), Br_2_, 7 h, darkness. (**m**) NaCl, 250 °C, 40 min; then AlCl_3_, 30 min. (**n**) compound (**3**)/DCM, HOBt, DMF, L-isoleucine methyl ester.

A series of eleven indanoyl derivatives structurally related to coronatine was synthesized (Figure 3), and its isoflavonoid-phytoalexin elicitor effect in hypocotyl-roots of the common bean was evaluated. All indanoyl derivatives were evaluated at 1.0 mM in hydroalcoholic solution (0.05%). Hypocotyl-roots treated with hydroalcoholic solution were used as control. The plant tissues were incubated for 72 h post-induction.

#### 2.1.1. Effect of C4-Substituted Indanoyl Derivatives (**4**, **9**, **10**, and **11**) on Phytoalexin Accumulation

**Figure 4:**
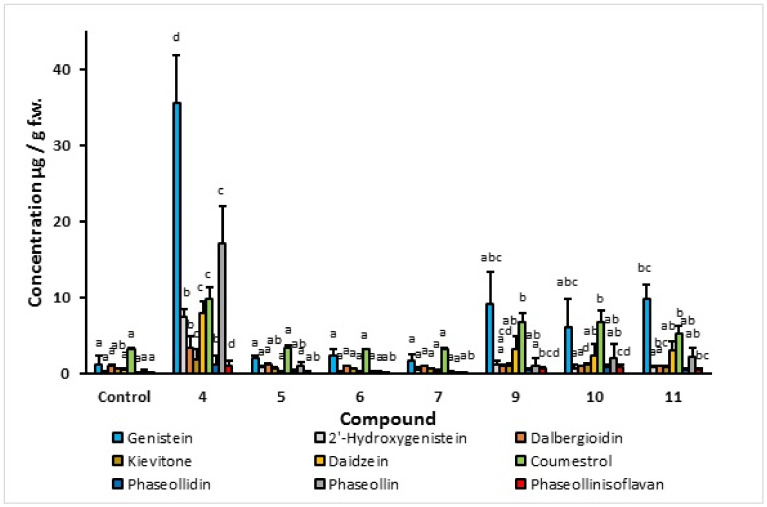
Isoflavonoid phytoalexin accumulation in hypocotyl-root of common bean (*P. vulgaris* L. cv. ICA Cerinza) in response to indanoyl-derivative treatment 72 h post-induction. Bars show the mean concentration of isoflavonoids ± s.d. (*n* = 3). For each compound, bars with different letters (a–d) are significantly different (*p* < 0.05; Fisher’s LSD test).

In order to select the most promising structural core as an elicitor, a screening based on the stimulation of the isoflavonoid phytoalexin production was carried out. A special interest was placed on the accumulation of phaseollin, a recognized antifungal substance biosynthesized in the later stages of the 5-deoxy isoflavonoid pathway. The results of isoflavonoid phytoalexin production in hypocotyl-roots of common bean (cv. ICA Cerinza) treated with an indanoyl ester (**9**), two indanoyl-alkyl amides (**10** and **11**), and an indanoyl-amino acid conjugate (**4**) are shown in Figure 4. Derivatives (**4**, **9**, **10**, and **11**) significantly increased the concentration of phytoalexins in comparison to the control (tissues treated with hydroalcoholic solution, 0.05%), particularly genistein, phaseollin, and coumestrol. In the cultivar ICA Cerinza, the highest accumulation of phytoalexins was elicited by the indanoyl-amino acid conjugate (**4**), with genistein (35.6 µg/g), phaseollin (17.7 µg/g), and coumestrol (10.0 µg/g) as the major phytoalexins. Remarkably, the amount of phaseollin in tissues treated with (**4**) was seventeen times greater than that found in the control. Similarly, in the cultivar Uribe Rosado, the highest accumulation of the phytoalexins genistein (39.2 µg/g) and phaseollin (21.9 µg/g) was observed when the tissues were treated with the indanoyl derivative (**4**), which is of interest because phaseollin and genistein have been reported as strong antifungal compounds [18,19], the production of which is directly related to resistance to phytopathogenic micro-organisms in common bean [20]. Even phaseollin has been proposed as a chemical marker of resistance to fungal diseases in common bean [13].

The concentration of phaseollin in the tissues treated with (**9**), (**10**), (**11**), and the control was below that found for daidzein and coumestrol. However, after treatment with (**4**), tissues exhibited amounts of phaseollin substantially higher than daidzein and coumestrol, which demonstrates that the branch of the biosynthetic pathway leading to the pterocarpans in common bean becomes more rapid and efficient as a result of the application of indanoyl derivative (**4**).

#### 2.1.2. Effect of Indanoyl-Amino Acid Conjugates (**4**, **5**, **6**, and **7**) on Phytoalexin Accumulation

As can be seen in Figure 4, the indanoyl-L-isoleucine methyl ester conjugate elicited the highest levels of phytoalexins, particularly phaseollin. Interestingly, the levels of phytoalexins found in the tissues of seedlings treated with (**5**), (**6**), and (**7**) were similar to those detected in the control (hydroalcoholic solution-treated seedlings). This shows that the indanoyl derivatives containing the amino acids L-leucine methyl ester, L-valine methyl ester, and L-glycine ester, respectively, were biologically inactive. The induction of phytoalexins in common bean with indanoyl derivatives appears to be highly dependent on structural features; even small differences in structure are sufficient to differentially elicit the phytoalexins. The indanoyl-amino acid conjugate containing the L-isoleucine methyl ester is of particularly interest because this structural element is easily derived from the amino acid building block coronamic acid of coronatine by opening the C(2)-C(3) bond of the cyclopropyl amino acid [8].

Studies with different indanoyl-amino acid conjugates have reported that only those containing isoleucine and leucine are biologically active in inducing defense volatiles in Lima bean (*P. lunatus* L.); the authors of these studies report that the loss of a –CH_2_– group, as in the case of valine, leads to a total loss of biological activity [9,21]. The above suggests that the interaction of the indanoyl elicitor with the receptor that triggers biological activity is very specific, discriminating small differences in the carbon chain of the amino acid or even between the L and D conformation [9]. Such an active site seems to be optimized for the isoleucine side chain, preferably by the L-conformation.

#### 2.1.3. Effect of Indanoyl-L-Isoleucine Methyl Ester Derivatives (**4**, **8**, **12**, **13**, and **14**) on Phytoalexin Accumulation

**Figure 5:**
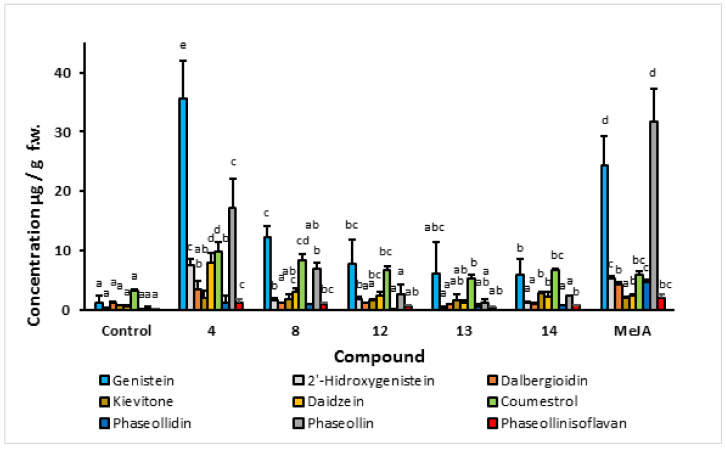
Isoflavonoid phytoalexin accumulation in hypocotyl-root of common bean (*P. vulgaris* L. cv. ICA Cerinza) in response to 1-oxo-indanoyl-L-isoleucyl methyl ester derivatives and MeJA treatments 72 h post-induction. Bars denote the mean concentration of isoflavonoids ± s.d. (*n* = 3). For each compound, bars with different letters (a–e) are significantly different (*p* < 0.05; Fisher’s LSD test).

Considering that previous studies showed compound (**4**) as a promising phytoalexin elicitor in common bean, it was used as a structural template to obtain new derivatives. Structural modifications included bromination at position 6 of (**2**) and condensation with the L-isoleucine methyl ester to obtain compound (**8**), reduction of the carbonyl group to obtain alcohol (**12**), aldol condensation at carbon 2 of (**2**) to yield the 2-benzylidene (**13**), and preparation of imine (**14**). As can be seen in Figure 5, treatment of tissues with the indanoyl-L-isoleucine methyl ester derivatives increased the concentration of phytoalexins in ICA Cerinza, particularly dalbergioidin, daidzein, 2′-hydroxygenistein, phaseollin, coumestrol, and kievitone. In the case of phaseollin, significant differences in concentration were detected between tissues treated with (**4**), (**8**), and (**12**) compared to the control. However, a significant loss of isoflavonoid phytoalexin elicitor activity was evident in both cultivars as a result of the carbonyl group reduction (at C1) (**13**) and the formation of hydrazone (**14**). Thus, for the cultivar Uribe Rosado, the concentration of phaseollin in hypocotyl-roots was decreased by twelve times and three times by treatment with (**13**) and (**14**), respectively, with respect to the tissues treated with (**4**). Therefore, the carbonyl group plays a very important role in the recognition of the derivative as a chemical signal for the activation of phytoalexin production and accumulation, which coincides with that reported in the specialized literature [8].

The derivative substituted with bromine at C-6 (**8**) exhibited an elicitor effect and a significant accumulation of 2′-hydroxygenistein, daidzein, genistein, coumestrol, and phaseollin compared to the control. However, its effect was lower than that observed for compound (**4**). Finally, the 2-benzylidene derivative (**13**) did not elicit phytoalexin accumulation in any of the common bean cultivars studied. This inactivity was accompanied by lower water solubility for this derivative. The low solubility limits the practical use of indanoyl derivatives, as mentioned in [21].

As a result of the systematic analysis of the effect of structure on phytoalexin elicitation in common bean hypocotyl-root, it was concluded that the most promising derivative corresponds to compound (**4**), which elicited an isoflavonoid accumulation similar to that exhibited by the known elicitor MeJA. Therefore, a more detailed analysis of the effect of concentration of (**4**) on phytoalexin-eliciting activity and over time was carried out.

### 2.2. Time-Course Experiments with 1-Oxo-Indanoyl-L-Isoleucyl Methyl Ester

**Figure 6:**
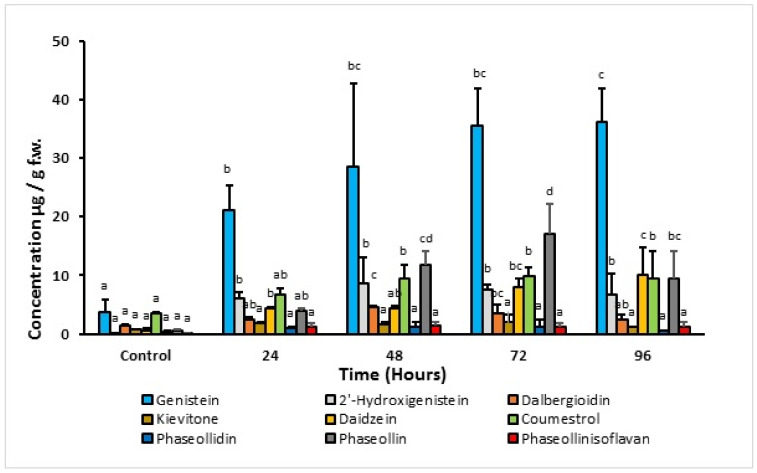
Time-course accumulation of phytoalexins in hypocotyl-root of common bean (*P. vulgaris* L. cv. ICA Cerinza) treated with 1.0 mM 1-oxo-indanoyl-L-isoleucyl methyl ester (**4**). Bars represent the mean concentrations of the isoflavonoids ± standard deviation (*n* = 3). For each compound, bars with different letters (a–d) are significantly different (*p* < 0.05; Fisher’s LSD test).

It is observed that the concentration of phytoalexins increases significantly with post-induction time compared to the control (Figure 6). During the first 48 h, in ICA Cerinza, maximum concentrations were detected for the isoflavones 2′-hydroxygenistein (9.0 μg/g) and the isoflavanone dalbergioidin (5.1 μg/g), after which the accumulation of the isoflavone and the isoflavanone decreased. Consequently, the concentration of coumestrol and pterocarpan-type phytoalexins increased, reaching maximum levels after 72 and 96 h post-induction, respectively. In ICA Cerinza, the maximum concentration of phaseollin (17.2 μg/g; 39 times higher than in the control) and coumestrol (9.8 μg/g) was reached at 72 h post-induction. Similar results were found in the cultivar Uribe Rosado; in particular, the maximum concentration of phaseollin (21.0 μg/g; 6.5 times higher than in the control) and coumestrol (15.9 μg/g; two times greater than in the control) was reached after 72 and 96 h post-induction, respectively. The amount of kievitone remained almost constant (below 5.0 μg/g) during the period of analysis for both cultivars.

The high concentrations of genistein in the two bean varieties but low levels of 2′-hydroxygenistein and elevated levels of dalbergioidin and kievitone clearly indicate that there is some metabolic blockage in the biosynthetic pathway of the 5-hydroxyisoflavonoids (genistein to 2′-hydroxygenistein to dalbergioidin to kievitone). Additionally, the low amount of kievitone may be the result of the tissue studied. Goossens et al. [22] found that the highest levels of this phytoalexin occur in the cotyledons and decrease in the hypocotyls and roots. In contrast, the 5-deoxyisoflavonoid biosynthetic pathway appears to be quite efficient in the tissues (hypocotyl-root) of both cultivars in response to treatment with 1-oxo-indanoyl-L-isoleucine methyl ester (**4**), as despite the low levels of the precursor daidzein detected, a relatively high concentration of phaseollin and coumestrol is elicited between 72 and 96 h post-induction. The low levels of phaseollidin clearly indicate that conversion to phaseollin is rapid (daidzein to phaseollidin to phaseollin). The metabolism of phaseollin to phaseollinisoflavan has been hypothesized to result from the need to reduce the phytotoxic effect of the phytoalexin phaseollin [23]. The low levels of phaseollinisoflavan detected as a result of elicitation with (**4**) suggest that the phaseollin concentrations achieved in both cultivars do not represent toxic levels to the tissues [23].

### 2.3. Phytotoxicity Effects of 1-Oxo-Indanoyl-L-Isoleucyl Methyl Ester on Bean Seedlings

It is important to evaluate the phytotoxic effects of elicitors on common bean, from seed germination to seedling growth, because a large number of negative effects has been reported [24]. Systematic analysis of the effects produced by the application of the synthesized elicitors at various stages of the seedling life cycle on common bean can yield information concerning the optimal conditions for the application and use of elicitors in the field. For this purpose, 1.0 mM 1-oxo-indanoyl-L-isoleucyl methyl ester (**4**), along with 1.0 mM MeJA and 0.05% ethanol, were separately applied over common bean seeds and seedlings, and their phytotoxic effects were evaluated. Seeds of the cultivar Uribe Rosado were used for the assays.

#### 2.3.1. Effect of 1-Oxo-Indanoyl-L-Isoleucyl Methyl Ester on Common Bean Seeds and Cotyledon Hardness

In a Petri dish on wet filter paper, forty common bean seeds were generously sprayed with the treatments daily for five days. The seed germination (%) and cotyledon hardness are presented in Table 1. After three days, the emerged radicle was evident. Interestingly, on the third day, the application of MeJA and (**4**) resulted in a significant increase in the percentage of germinated seeds: 56.9 and 71.7%, respectively. For the remaining days, no significant differences in seed germination percentage were observed. It has been shown that treatment with some elicitors, such as salicylic or acetylsalicylic acid, significantly improved the germination percentages in carrot seeds [25].

On day 5, the length and number of secondary roots were measured. The results showed no significant differences in the number of roots and their length between the water-treated seedlings and those with 1.0 mM MeJA. However, significant differences were evident with the application of (**4**), resulting in a reduction in the length of roots, a total inhibition in the growth of secondary roots, and a reduction in the hardness of cotyledons. These effects on root length and growth have been documented in studies of coronalon (an indanoyl derivative) analogs [8]. Similarly, a significant reduction in the hardness of cotyledons was observed with the application of (**4**), possibly due to histological and structural changes and differences in the activation of the enzymatic machinery of the common bean seeds [26]. Rao et al. [27] studied the effect of the application of coronatine and MeJA on tomatoes, observing changes in chloroplast structure, cell wall thickening, accumulation of proteinase inhibitors, induction of anthocyanins, and root growth inhibition.

#### 2.3.2. Effect of 1-Oxo-Indanoyl-L-Isoleucyl Methyl Ester on Common Bean Seedlings and Chlorophyll Content

Important variables related to the physiology and functioning of the seedlings and roots were monitored, such as seedling size, stem size, leaf size, length, number of secondary roots (Table 2), and chlorophyll content. The results show that there was no significant difference in the variables studied for days 7, 9, 11, and 13. Considering the above results, it is observed that the spraying of 1.0 mM of derivative (**4**) on common bean seedlings had no significant effect on hypocotyls and roots during this stage of development. The above results and the inhibition of secondary root growth in the germination stage may suggest that it is more convenient to apply derivative (**4**) during a later stage after seed germination. Other authors have found negative effects on seedling, hypocotyl, and leaf size after exogenous application of MeJA in common bean and lupin, respectively [28,29].

The exogenous application of cinnamic acid derivatives in common bean [28] and salicylic acid in barley [24] have shown that as the concentration of the derivative increases, there is a greater inhibitory effect on seedling root growth. In the case of salicylic acid, which is known to be a plant growth regulator, it was observed that a low concentration increased root size, but at high concentrations, the inhibition of growth was significant.

On the other hand, chlorophyll content is a very important variable in the normal functioning of seedlings due to its participation in the photosynthesis process; therefore, on day 13, chlorophyll content was measured, taking into account the reports of [30]. The chlorophyll contents in leaves of common bean seedlings after treatments with 0.05% ethanol, 1.0 mM MeJA, and 1.0 mM (**4**) were 142.6 ± 37.2, 181.0 ± 35.7, and 126.3 ± 20.9 mg/g fresh weight, respectively. The obtained results do not show significant differences in chlorophyll content after applying the elicitor solutions for 8 days. In addition, no color changes or signs of chlorosis were observed in the leaves of the common bean seedlings during treatment with the elicitors. In contrast with these results, Pancheva et al. [24] observed a decrease in the amount of chlorophylls produced in wheat seedlings with the application of salicylic acid, where the amount of chlorophylls was concentration- and contact-time-dependent. Likewise, Xie et al. [31] reported a reduction in chlorophyll production in cotton seedlings as a result of treatment with coronatine. Therefore, the low phytotoxicity and the strong isoflavonoid phytoalexin elicitor effect convert to indanoyl-type synthetic compound (**4**) in a promising option in the long or medium term as an agronomic alternative for disease control in common bean.

## 3. Discussion

Stimulating the natural defense mechanisms of plants, such as phytoalexins, through the application of elicitors offers new alternatives for disease control in crops of importance [13]. Therefore, it is important to continue with the design of new elicitors that allow for greater diversity and the generation of knowledge about plant–elicitor interactions, as well as the main factors that influence this response [3]. Indanoyl elicitors can modify the chemical response of common bean seedlings in Colombian cultivars, such as ICA Cerinza and Uribe Rosado, generating a mixture of phytoalexins with possible potential for non-biocidal control of diseases, such as anthracnose. However, the search for new elicitors should not focus only on phytoalexin accumulation studies; it is also necessary to evaluate the effects on the growth and development of the seedlings treated with the elicitor, which will allow for progress towards the application of elicitors with greater potential in crops. In this work, the elicitor process, as well as phytotoxicity, of 1-oxo-indanoyl-L-isoleucine methyl ester was evaluated in bean seedlings of two Colombian bean varieties.

(i) Structure of the elicitor. Systematic modifications of the 1-oxo-indane-4-carboxylic acid system showed the importance of different functional groups and substituents in inducing activity of isoflavonoid phytoalexins. First, a change at position 4 by another substituent different from the amino acid L-isoleucine methyl ester resulted in a significant loss of the abovementioned biological activity; second, the carbonyl group of the oxo-cyclopentyl system plays an important role in the elicitor activity; reduction of this group or nucleophilic addition for hydrazone formation had negative effects on the activity. Finally, incorporation of a bulky substituent, such as bromine, at position 6 significantly reduced the elicitor activity. It is clear that it is possible to modulate phytoalexin production in beans with the use of indanoyl-type elicitors.
(ii) Elicitation process. Phytoalexin accumulation depends on the common bean cultivar, the post-induction time, and the elicitor structure. The maximum concentrations of isoflavonoid phytoalexins were elicited after treatment with the compound 1-oxo-indanoyl-L-isoleucine methyl ester (**4**) and a post-induction time of 72 h. Additionally, the major phytoalexins in the elicitation process were genistein and phaseollin, which are recognized for their antimicrobial effect. Likewise, the application of indanoyl derivatives showed that the 5-deoxyisoflavonoid production pathway, which uses daidzein as a precursor, was preferentially channeled to the pterocarpans biosynthetic pathway, specifically phaseollin. In the case of the 5-hydroxyisoflavonoid biosynthetic pathway, a high concentration of genistein was observed but a low concentration of 2′-hydroxygenistein, with considerably reduced levels of dalbergioidin and kievitone; this may suggest that the 5-hydroxyisoflavonoid phytoalexin pathway is repressed or blocked in the tissues of the cultivars ICA Cerinza and Uribe Rosado.
(iii) Phytotoxicity test. The compound 1-oxo-indanoyl-L-isoleucine methyl ester exhibited a low phytotoxic effect on different bean tissues. The elicitor should be applied during a later stage after germination because inhibition of root growth was evidenced. The insignificant or null effects on seedling size, stem and leaves, chlorophyll content, number of secondary roots, and root length, along with the strong phytoalexin-elicitor capacity of 1-oxo-indanoyl-L-isoleucine methyl ester, make it a potential elicitor for disease control that could be applied in the field.

## 4. Materials and Methods

### 4.1. General Methods

Column chromatography (CC) was performed using silica gel 60 (0.040–0.063 mm, Merck, Darmstadt, Germany) and/or Sephadex LH20 (Sigma-Aldrich, St. Louis, MO, USA) as stationary phases. Thin-layer chromatography (TLC) was performed on aluminum plates precoated with silica gel (Si 60 F254, 0.25 mm, Merck, Darmstadt, Germany) using mixtures of *n*-hexane/ethyl acetate (EtOAc) as mobile phase. Visualization was carried out with UV radiation (254 and 365 nm) (UVP UVGL-58) and by spraying with acetic acid (AcOH)/sulfuric acid/water (143:28:30) (oleum) or Dragendorff’s reagent, followed by heating. Liquid chromatography (LC) analyses were carried out using a Shimadzu Prominence LC 20AT chromatograph with autosampler (Shimadzu, Kyoto, Japan), diode array detector (DAD), and column thermostat. Structural elucidation was performed using 1D and 2D nuclear magnetic resonance (NMR) experiments on a Bruker AMX 300 spectrometer (Bruker, Germany; 300 and 75 MHz for ^1^H and ^13^C, respectively) using methanol-d4 and CDCl_3_ as solvent. Coupling constants (J) are in Hertz (Hz), and chemical shifts (δ) are expressed in parts per million (ppm) and reported relative to residual solvent peaks. Infrared spectra were recorded on a Shimadzu Tracer-100 FT-IR spectrometer in the range of 4000–400 cm^−1^. The ultraviolet (UV) spectra were obtained on a Shimadzu UV-1800 spectrophotometer.

### 4.2. Chemicals

L-isoleucine methyl ester, L-leucine methyl ester, 3-(dimethylamino)propylamine, 3-(diethylamino)propylamine, benzylhydrazine, 3-bromobenzaldehyde, 3-bromo-1-propanol, MeJA, palladium on carbon, *N*,*N*′-dicyclohexylcarbodiimide (DCC), 1-hydroxybenzotriazole (HOBt), *N*,*N*-dimethylformamide (DMF), genistein, daidzein, and coumestrol were purchased from Sigma-Aldrich (St. Louis, MO, USA). 2-carboxycinnamic acid, L-valine methyl ester, glycine methyl ester, and anhydrous aluminum chloride (AlCl_3_) were obtained from Alfa Aesar Co. (Ward Hill, MA, USA). 2′-hidroxygenistein, dalbergioidin, kievitone, phaseollidin, phaseollinisoflavan, and phaseollin were isolated from a previous work described in [1]. Dichloromethane (DCM), ethyl acetate (EtOAc), and *n*-hexane (*n*-hex) were acquired from Protokimica (Medellín, Colombia) and doubly distilled. Solvents for HPLC were obtained from Merck (Darmstadt, Germany). Ultrapure water (type 1 and 2) was produced by Synergy^®^ UV and Elix Technology (Millipore, Merck) water purification systems (Darmstadt, Germany). 1-oxo-indane-4-carboxylic acid was prepared according to processes described in [9] and [13].

### 4.3. Plant Material

Common bean seeds (*P. vulgaris* L.), cultivars ICA Cerinza and Uribe Rosado, were purchased from Semicol Ltd.a. (Bogotá, Colombia) and Semillas & Semillas Ltd.a. (Medellín, Colombia), respectively. ICA Cerinza is tolerant to anthracnose (*C. lindemuthianum* Sacc.), rust (*Uromyces phaseoli*), oidium (*Erysiphe potygoni* DC), and Fusarium root rot (*Fusarium* sp.) [32]. Uribe Rosado is susceptible to diseases, including anthracnose [33]. Seeds were immersed for 5 min in sodium hypochlorite (NaClO, 5%) for sterilization and further washed with distilled water. The seeds were sown in quartz sand and stored under room-temperature conditions in the dark. After 5 days, the seedlings were uprooted and used in the elicitation experiments.

### 4.4. Synthesis and Identification of Indanoyl Derivatives

2-carboxyhidrocinnamic acid was obtained by hydrogenation using palladium on carbon as catalyst from 2-carboxycinnamic acid (**1**) (yield: 99%). Sodium chloride (NaCl) and anhydrous AlCl_3_ were then used to perform an intramolecular Friedel–Crafts cyclization of 2-carboxyhydrocinnamic acid, which produced the indanone skeleton [13] (yield (**2**): 63%). Additionally, bromination of 2-carboxyhidrocinnamic acid using bromine (Br_2_) in concentrated nitric acid (HNO_3_) was carried out to afford 6-bromo-1-oxo-indane-4-carboxylic acid after purification by chromatography column [34]. Next, 1-oxo-indane-4-carboxylic acid (**2**) or 6-bromo-1-oxo-indane-4-carboxylic acid (**3**) was subjected to a coupling reaction with the methyl esters of different amino acids, DCC and HOBt, for the synthesis of indanoyl amino acid conjugates (**4**, **5**, **6**, **7**, and **8**) [9]. In the same way, some indanoyl amides (**9**, **10**) were obtained using DCC and HOBt. An indanoyl ester (**11**) was prepared by reaction between (**2**) and an alkyl halide in the presence of DMF. Additionally, 2-benzylidene (**13**) and imine (**14**) were obtained using conventional reactions (cross-Aldol condensation and imine formation, respectively).

#### 4.4.1. Indanoyl-Amino Acid Conjugates and Indanoyl Amides

1-oxo-indane-4-carboxylic acid (**2**, 1.0 eq) or 6-bromo-1-oxo-indane-4-carboxylic acid (**3**, 1.0 eq), HOBt (1.5 eq), DMF (1.0 eq), and the methyl esters of the amino acids (1.0 eq) or the alkyl amines [3-(dimethylamino)-1-propylamine or 3-(diethylamino)-1-propylamine] were dissolved in DCM at room temperature and prepared according to the process described in [13]. The resulting products were subjected to purification by column chromatography using silica gel (mobile phase: mixtures *n*-hex:EtOAc from 7:3 to 2:8 *v*/*v* for indanoyl-amino acid conjugates, and DCM:methanol, 9:1 to 1:1 *v*/*v* for indanoyl amides). Then, a second stage of purification was necessary using Sephadex LH-20 (mixture *n*-hex:DCM:methanol, 2:1:1, *v*/*v*). Derivatives (**4**), (**5**), (**6**), (**7**), and (**8**) were obtained as solids. Spectroscopic data for indanoyl-amino acid conjugates (**4**), (**5**), and (**6**) coincide with those reported in [9], and the HRMS data were 304.1543 (calcd for C_17_H_21_NO_4_ [M + H]^+^: 304.1543), 304.1539 (calcd for C_17_H_21_NO_4_ [M + H]^+^: 304.1543) and 290.1385 (calcd for C_16_H_19_NO_4_ [M + H]^+^: 290.1386), respectively. Spectroscopic data for (**7**), (**8**), and indanoyl amides are presented as follows: *1-oxo-indanoyl-glycyl methyl ester* (**7**) (Yield: 19%). M. p. = 105 °C. ^1^H NMR (CDCl_3_, 300 MHz): 2.73 (2H, m, H2), 3.45 (2H, t, *J* = 5.7 Hz, H3), 3.84 (3H, s, –OCH_3_), 4.3 (2H, d, *J* = 5.1 Hz, H2′), 6.79 (NH), 7.48 (1H, t, *J* = 7.5 Hz, H6), 7.89 (2H, d, *J* = 3.6 Hz, *J* = 7.5 Hz, H7 and H5). ^13^C NMR (CDCl_3_, 75 MHz): 26.1 (C3), 36.2 (C2), 41.6 (C2′), 52.65 (–OCH_3_), 126.6 (C7), 127.7 (C6), 132.5 (C5), 132.7 (C4), 138.3 (C7a), 154.4 (C3a), 167.1 (–CONH), 170.4 (–COO–), 206.5 (C1). MS (ESI) *m*/*z*: calcd for C_13_H_13_NO_4_ [M + H]^+^: 248.0917. Found: 248.0916. *6-bromo-1-oxo-indanoyl-L-isoleucyl methyl ester* (**8**) (Yield: 59%). ^1^H NMR (CDCl_3_, 300 MHz): 1.05 (6H, m, H5′, H6′), 1.32 (1H, m, H4′), 1.55 (1H, m, H4′), 2.08 (1H, m, H3′), 2.78 (2H, t, *J* = 6.0, H2), 3.39 (2H, m, H3), 3.85 (3H, s, –OCH_3_), 4.86 (1H, dd, *J* = 4.5 H2′), 6.65 (NH), 7.98 (1H, d, *J* = 1.5, H7), 8.02 (1H, d, *J* = 1.2, H5). ^13^C NMR (CDCl_3_, 75 MHz): 11.7 (C5′), 15.6 (C6′), 25.4 (C3), 25.8 (C4′), 36.3 (C2), 38.2 (C3′), 52.5 (–OCH_3_), 56.9 (C2′), 121.7 (C6), 129.3 (C7), 134.6 (C4), 135.5 (C5), 140.1 (C7a), 152.4 (C3a), 165.3 (–CONH), 172.4 (–COO), 204.81 (C1). *N-(3-(dimethylamine)propyl)-1-oxo-indane-4-carboxamide* (**9**) (Yield: 22%). Amorphous solid. M. p. = 218 °C. TLC: Rf (DCM:methanol, 8:2); 0.29 (red spot with Dragendorff’s reagent). ^1^H NMR (CDCl_3_, 300 MHz): 1.93 (2H, m, H2′), 2.42 (6H, s, H4′, H5′), 2.67 (3H, t, *J* = 6.0, H3′), 2.75 (2H, m, *J* = 4.2, H3), 3.52 (3H, t, *J* = 6.0, H2), 3.62 (3H, m, *J* = 5.1, H1′), 7.48 (1H, t, *J* = 7.5, H6), 7.88 (2H, t, *J* = 8.1, H5, H7), 8.39 (–NH–). ^13^C NMR (CDCl_3_, 75 MHz): 26.3 (C2), 34.0 (C2′), 36.3 (C3), 39.8 (C1′), 45.0 (C4′, C5′), 58.6 (C3′), 126.1 (C7), 127.6 (C6), 132.4 (C5), 133.3 (C4), 138.3 (C7a), 154.7 (C3a), 167.0 (–NHCO–), 207.0 (C1). MS (ESI) *m*/*z*: calcd for C_15_H_20_N_2_O_2_ [M + H]^+^: 261.1597. Found: 261.1594. *N-(3-(diethylamine)propyl)-1-oxo-indane-4-carboxamide* (**10**) (Yield: 35%). Colorless oil. TLC: Rf (DCM:methanol, 8:2); 0.37 (red spot with Dragendorff’s reagent). ^1^H NMR (CD_3_OD, 300 MHz): 1.09 (6H, t, H5′, H7′), 1.84 (2H, m, H2′), 2.62–2.66 (2H, m, H4′, H6′), 2.69 (2H, m, H2), 3.32 (2H, t, *J* =1.5, H3′), 3.37 (2H, s, H3), 3.44 (2H, t, *J* = 6.9, H1′), 7.52 (1H, s, H6), 7.86 (2H, t, *J* = 9.3, H5, H7). ^13^C NMR (CD_3_OD, 75 MHz): 9.7 (C5′, C7′), 25.2 (C2′, C3′), 25.5 (C3), 37.9 (C1′), 46.3 (C4′, C6′), 49.9 (C2), 125.3 (C7), 127.4 (C6), 132.7 (C5), 133.9 (C4), 137.7 (C7a), 154.3 (C3a), 168.6 (–NHCO–), 207.7 (C1). MS (ESI) *m*/*z*: calcd for C_17_H_24_N_2_O_2_ [M + H]^+^: 289.1910. Found: 289.1908

#### 4.4.2. Indanoyl Ester

The derivate was prepared starting from 1-oxo-indane-4-carboxylic acid (**2**) according to the process described in [13]. The resulting product was subjected to purification by column chromatography using silica gel (mobile phase: mixture *n*-hex:EtOAc 1:1, *v*/*v*). Spectroscopic data for the indanoyl ester are presented as follows: *3′-hydroxypropyl 1-oxo-indane-4-carboxylate* (**11**) (Yield: 53%). Yellow solid. M. p. = 150 °C. TLC: Rf (*n*-hex:EtOAc, 7:3); 0.19 (yellow spot with oleum). ^1^H NMR (CDCl_3_, 300 MHz): 2.07 (2H, t, *J* = 5.7, H2′), 2.32 (–OH), 2.74 (2H, m, H2), 3.5 (2H, t, *J* = 5.7, H3), 3.84 (2H, t, *J* = 6.0, H3′), 4.55 (2H, t, *J* = 6.0, H1′), 7.5 (1H, m, H6), 7.97 (1H, d, *J* = 7.5, H6), 8.31 (1H, d, *J* = 7.8, H5). ^13^C NMR (CDCl_3_, 75 MHz): 27.3 (C3), 31.8 (C2′), 36.1 (C2), 59.1 (C3′), 62.1 (C1′), 127.6 (C6), 128.2 (C4), 128.2 (C7), 136.4 (C5), 138.3 (C7a), 156.7 (C3a), 166.1 (–COO–), 206.7 (C1).

#### 4.4.3. Carbonyl Group Reduction

1-oxo-indanoyl-L-isoleucyl methyl ester (**4**) was reduced using NaBH_4_ dissolved in a mixture of methanol and dry DCM, 2:3, *v*/*v* according to [9]. *1-hydroxy-indanol-L-isoleucyl methyl ester* (**12**) (Yield: 52%). White solid. M.p. = 80 °C. TLC: Rf (*n*-hex:EtOAc, 7:3); 0.38 (yellow spot with oleum). IR (cm^−1^): 3313, 3278, 1716, 1633. ^1^H NMR (CDCl_3_, 300 MHz): 1.0 (6H, m, H5′, H6′), 1.25 (1H, m, H4′), 1.52 (1H, m, H4′), 2.03 (2H, m, *J* = 1.8, H2), 2.51 (1H, m, *J* = 4.8, H3′), 2.69 (–OH), 3,03 (1H, m, H3), 3.26 (1H, m, H3), 3.80 (3H, s, –OCH_3_), 4.82 (1H, m, *J* = 4.8, H2′), 5.23 (1H, s, H1), 6.59 (NH), 7.30 (1H, t, *J* = 6.0, H6), 7.55 (1H, m, *J* = 7.5, H7), 7.57 (1H, m, *J* = 7.5, H5). ^13^C NMR (CDCl_3_, 75 MHz): 11.6 (C5′), 15.6 (C6′), 25.3 (C4′), 29.9 (C3), 35.8 (C2), 38.2 (C3′), 52.3 (–OCH_3_), 56.7 (C2′), 75.7 (C1), 127.1 (C7), 127.2 (C6), 131.5 (C5), 142.0 (C4), 146.8 (C7a), 168.0 (–COO–), 172.0 (C1′). MS (ESI) *m*/*z*: calcd for C_17_H_23_NO_4_ [M + H]^+^: 306.1699. Found: 306.1696.

#### 4.4.4. 2-Benzylidene

A few drops of piperidine were added to (**4**); the mixture was stirred manually for 1 min, after which 1.5 equivalents of 3-bromobenzaldehyde were added. The reaction was stirred periodically at room temperature for 12 h. The product was obtained as an insoluble solid; it was recrystallized with *n*-hex-DCM-methanol mixture, 2:1:1, *v*/*v* and filtered. *2-(3′-bromobenzylidene)-1-oxo-indanoyl-L-isoleucyl methyl ester* (**13**) (Yield: 33%). Light green solid. M.p. = 198 °C. TLC: Rf (*n*-hex:EtOAc, 7:3); 0.46 (yellow spot with oleum). ^1^H NMR (CDCl_3_, 300 MHz): 1.05 (6H, t, H5′, H7′), 1.10 (1H, m, H4′), 1.98 (1H, dd, H4′), 3.51 (1H, t, H3′), 3.89 (3H, s, –OCH_3_), 4.36 (2H, m, H3), 4.92 (1H, m, H2′), 6.73 (1H, –NH–), 7.41 (1H, d, *J* = 8.1 Hz, H7), 7.56 (1H, m, H2″), 7.61 (1H, m, H6″), 7.64 (1H, s, Ha), 7.71 (1H, d, H5″), 7.84 (1H, s, H5), 7.95 (1H, t, H4″), 8.07 (1H, d, *J* = 7.5 Hz, H6). ^13^C NMR (CDCl_3_, 75 MHz): 11.7 (C5′), 15.7 (C6′), 33.9 (C3), 35.6 (C4′), 49.2 (C3′), 53.0 (–OCH_3_), 56.9 (C2′), 123.1 (C3″), 127.3 (C6), 128.2 (C6″), 128.9 (C5″), 130.5 (C7), 132.3 (C4), 132.5 (C4″), 132.7 (C2″), 133.1 (Ca), 133.8 (C5), 135.4 (C4), 137.2 (C1″), 148.9 (C7a), 156.8 (C3a), 166.6 (–NHCO–), 172.5 (–COO–), 193.5 (C1). MS (ESI) *m*/*z*: calcd for C_24_H_24_BrNO_4_ [M + H]^+^: 470.0961. Found: 470.0964.

#### 4.4.5. Imine

Benzylhydrazine (1.5 equivalents) was added to (**4**) dissolved in ethanol. The reaction was stirred at reflux for 24 h. The resulting product was subjected to two stages of purification by column chromatography, first using a Sephadex LH-20 (mixture *n*-hex:DCM:methanol, 2:1:1, *v*/*v*) and then using silica gel (mobile phase: chloroform). *1-benzylhydrazono-indanoyl-L-isoleucyl methyl ester* (**14**) (Yield: 37%). White solid. M. p. = 65 °C. TLC: Rf (*n*-hex:EtOAc, 7:3); 0.39 (yellow spot with oleum). ^1^H NMR (CD_3_OD, 300 MHz): 1.05 (6H, m, H5′, H6′), 1.31–1.39 (1H, m, H4′), 1.5–1.61 (1H, m, H4′), 2.00–2.05 (1H, m, H3′), 3.03–3.07 (2H, m, H2), 3.32 (2H, d, *J* = 1.5 Hz, H3), 3.36 (2H, s, Ha), 3.78 (–OCH_3_), 4.58 (1H, m, H2′), 7.43 (1H, m, H6), 7.48–7.56 (2H, m, H3″, H5″), 7.60 (1H, m, H4″), 7.68 (1H, m, H5), 7.91 (2H, d, *J* = 6.9 Hz, H2″, H6″), 8.14 (1H, d, *J* = 7.8 Hz, H7). ^13^C NMR (CD_3_OD, 75 MHz): 10.2 (C5′), 14.7 (C6′), 25.3 (C4′), 27.0 (C2), 28.1 (C3), 36.7 (C3′), 48.5 (Ca), 51.2 (–OCH_3_), 57.4 (C2′), 124.8 (C7), 127.2 (C6), 127.5 (C3″, C5″), 128.3 (C2″, C6″), 129.7 (C5), 131.8 (C4″), 132.5 (C4), 133.1 (C3a), 138.6 (C7a), 147.9 (C1″), 164.7 (C1), 169.6 (–NHCO–), 172.4 (–COO–).

### 4.5. Induction Experiments

#### 4.5.1. Inducer Effect of Indanoyl Derivatives

Common bean seedlings (average height of 4 to 6 cm) cvs. ICA Cerinza and Uribe Rosado were immersed for 3 h in 1.0 mM of the 1-oxo-indanoyl derivatives (4 to 14) or 1.0 mM MeJA (positive control). A low concentration of ethanol (0.05%, *v*/*v*) was used to improve the solubility of each compound in water. As a negative control, common bean seedlings were submerged in a hydroalcoholic solution (0.05%). Plant material was then transferred to sterilized plastic containers and sown on moist cellucotton in the dark at 25 °C for 72 h. Subsequently, cotyledons, epicotyls, and hypocotyls-roots were carefully separated. The cotyledons and epicotyls were discarded, and the hypocotyls-roots (5.0 g) were used for elicitation analysis. All experiments were tested three times.

#### 4.5.2. Time-Course Experiments

Common bean seedlings (average height of 4 to 6 cm) cvs. ICA Cerinza and Uribe Rosado were immersed for 3 h in hydroalcoholic solution at 1.00 mM of the elicitor that generated the highest phytoalexin accumulation. In plastic containers, the plant material was sown in moist cellucotton and stored for 24, 48, 72, and 96 h (post-induction times) at room temperature in the dark. Hypocotyls-roots (5 g) were separated and analyzed after extraction [2]. Each experiment was conducted in triplicate.

### 4.6. Extraction, Detection, and Quantification of Isoflavonoid Compounds

Hypocotyl-root samples were processed according to [2]. A chromatograph (Prominence LC 20AT, Shimadzu) equipped with a diode array detector (DAD) was used to analyze the samples according to [13]. Methanol and water (0.05% acetic acid) were used as solvents. The mobile phase was from 10 to 70% methanol in 40 min, then 70 to 90% methanol in 20 min, followed by 10% methanol in 3 min and re-equilibration of the column in 8 min. A Luna C18 column (5 μm, 150 mm × 4.6 mm i.d.) at 30 °C, with a guard column Phenomenex ODS (4 mm × 3.0 mm i.d.) and a flow rate of 0.7 mL/min were used for the separation of isoflavonoids. A sample volume of 20 μL was injected. Detection of isoflavonoid compounds was performed at wavelengths of 248, 259, 278, 286, and 343 nm. Results are expressed as μg isoflavonoid/g fresh material (g f.w.).

### 4.7. Phytotoxicity Tests

The phytotoxic effect on common bean of the elicitor that generated the highest phytoalexin accumulation was evaluated at a concentration of 1.0 mM. In addition, MeJA (1.0 mM) and ethanol (0.05%, *v*/*v*) were used as positive and negative control, respectively, for comparison purposes. Tests were carried out on the cultivar Uribe Rosado, analyzing seed germination, cotyledon hardness, and seedling and root growth after regular and periodic spraying with the elicitor or ethanol (0.05%, *v*/*v*). Eight seeds were used in each plate, and tests were performed in quintuplicate.

#### 4.7.1. Assays on Common Bean Seeds and Cotyledons Hardness

For the evaluation of seed germination, the methodology reported in [28] was used, with some modifications. In a Petri dish on wet filter paper, 1.0 mM indanoyl derivative, 1.0 mM MeJA, or 0.05% ethanol was applied daily to forty seeds. On the fifth day of growth, the tegument was carefully removed; the presence or absence of secondary roots was examined, and the length of radicles was measured. In addition, cotyledons were used to measure their hardness. Puncture and compression tests were performed on cotyledons according to [35].

#### 4.7.2. Assays on Common Bean Seedlings and Chlorophyll Content

Common bean seedlings (cv. Uribe Rosado) with 5-day growth were generously sprayed with the treatments (1.0 mM indanoyl derivative, 1.0 mM MeJA, or 0.05% ethanol). This process was repeated every two days for one week. On the non-treatment days, seedlings were sprayed with water to avoid wilting. On the seventh day, seedlings were removed from the vermiculite; root, seedling, stem, and leaf size, along with the number of secondary roots and leaf coloration, were measured. Seedlings were exposed to diffused light at room temperature and 80% relative humidity. Chlorophyll content: fresh leaves (200 mg) were extracted with an aqueous solution of ethanol (80%) for 20 min in a water bath at 80 °C. The mixture was then filtered, and the filtrate was adjusted to a volume of 5 mL. The absorbance of the solution was measured at 654 nm in a spectrophotometer, and the chlorophyll content was calculated by taking into account the extinction coefficient of chlorophyll. The results were expressed as mg/g fresh weight [36].

### 4.8. Statistical Analysis

Values are expressed as mean ± standard deviation (s.d.). Data within the groups were analyzed using one-way analysis of variance (ANOVA), followed by Fischer’s least significant differences (LSD) test at *p* ≤ 0.05.

## 5. Conclusions

The accumulation of isoflavonoid phytoalexins in common bean tissues was found to depend on the time after induction and the structure of the elicitor. During the first 48 h, the biosynthetic precursors, isoflavones and isoflavanones, were the main phytoalexins; then, coumestrol and pterocarpan-type phytoalexins increased, reaching maximum levels. A metabolic blockage in the biosynthetic pathway in the 5-hydroxyisoflavonoid biosynthetic pathway could be suggested. Analysis of a series of eleven indanoyl derivatives revealed that some structural requirements are necessary for the elicitor effect. Variations in the C-4 or C-6 position of the indanoyl system and the carbonyl group of the oxo-cyclopentyl system resulted in significant changes in activity. The compound 1-oxo-indanoyl-L-isoleucine methyl ester is a promising elicitor, stimulating the synthesis and accumulation of phytoalexins in common bean tissues without significant phytotoxic effects on seeds and seedlings.

## Figures and Tables

**Table 1:** Effect of spraying with 1-oxo-indanoyl-L-isoleucyl methyl ester (**4**) and MeJA on seed germination (%), cotyledon hardness (puncture and compression tests), and radicle number and length of common bean (*P. vulgaris* L. cv. Uribe Rosado). All values are presented as mean ± standard deviation (*n* = 3); for each trial, eight seeds were used.

	% Seed Germination			Force (N)		Roots	
Treatment	Day 3	Day 4	Day 5	Puncture	Compression	Radicle Length (cm)	Number of Secondary Roots
**Negative Control**	21.0 ± 11.9 ^a^	95.5 ± 6.2 ^a^	97.5 ± 5.6 ^a^	10.6 ± 1.0 ^b^	167.2 ± 21.9 ^b^	6.7 ± 1.2 ^b^	11.3 ± 2.7 ^b^
**MeJA**	56.9 ± 21.1 ^b^	81.1 ± 15.9 ^a^	92.8 ± 11.0 ^a^	10.9 ± 1.3 ^b^	152.2 ± 36.8 ^b^	6.3 ± 0.8 ^b^	10.1 ± 1.8 ^b^
**(4)**	71.7 ± 21.6 ^b^	90.0 ± 16.3 ^a^	100.0 ± 0.0 ^a^	8.8 ± 1.2 ^a^	114.5 ± 19.1 ^a^	4.6 ± 0.5 ^a^	1.9 ± 1.8 ^a^

Different letters (^a, b^) in the same column indicate a significant difference at *p* < 0.05 (Fisher’s LSD test) (*n* = 8).

**Table 2:** Effect of spraying with 1-oxo-indanoyl-L-isoleucyl methyl ester (**4**) and MeJA on growth of common bean seedlings (*P. vulgaris* L. cv. Uribe Rosado). Ten seedlings were used each day; all values are presented as mean ± standard deviation.

	Day 9			Day 13		
Treatment	Plant Size (cm)	Hypocotyl Size (cm)	Leaf Size (cm)	Plant Size (cm)	Hypocotyl Size (cm)	Leaf Size (cm)
**Negative Control**	38.0 ± 1.0 ^a^	19.3 ± 1.2 ^a^	5.7 ± 0.8 ^a^	38.0 ± 1.0 ^a^	21.8 ± 0.8 ^a^	6.7 ± 0.3 ^a^
**MeJA**	36.0 ± 2.0 ^a^	18.2 ± 1.0 ^a^	6.0 ± 0.5 ^a^	38.2 ± 0.8 ^a^	19.0 ± 1.0 ^a^	6.3 ± 0.3 ^a^
**(4)**	35.7 ± 1.5 ^a^	17.3 ± 0.6 ^a^	5.3 ± 0.3 ^a^	38.2 ± 1.0 ^a^	19.2 ± 1.3 ^a^	6.2 ± 0.3 ^a^

Similar letter (^a^) in the same column indicates non-significant difference at *p* < 0.05 (Fisher’s LSD test) (*n* = 10).

## Data Availability

Data are available upon request to the Corresponding Authors.

## Sample Availability

Samples of the indanoyl derivatives are available from the authors.
